# Assessment of the impact of implementation research on the Visceral Leishmaniasis (VL) elimination efforts in Nepal

**DOI:** 10.1371/journal.pntd.0011714

**Published:** 2023-11-09

**Authors:** Anand Ballabh Joshi, Megha Raj Banjara, Sachi Chuke, Axel Kroeger, Saurabh Jain, Abraham Aseffa, John C. Reeder

**Affiliations:** 1 Public Health and Infectious Disease Research Center (PHIDReC), Kathmandu, Nepal; 2 Central Department of Microbiology, Tribhuvan University, Kirtipur, Kathmandu, Nepal; 3 UNICEF/UNDP/World Bank/WHO Special Programme for Research and Training in Tropical Diseases (TDR), Geneva, Switzerland; 4 Freiburg University, Centre for Medicine and Society, Freiburg, Germany; 5 Department of Control of Neglected Tropical Diseases, WHO, Geneva, Switzerland; Institute of Postgraduate Medical Education and Research, INDIA

## Abstract

Nepal, Bangladesh, and India signed a Memorandum of Understanding (MoU) in 2005 to eliminate visceral leishmaniasis (VL) as a public health problem from the Indian subcontinent by 2015. By 2021, the number of reported VL cases in these countries had declined by over 95% compared to 2007. This dramatic success was achieved through an elimination programme that implemented early case detection and effective treatment, vector control, disease surveillance, community participation, and operational research that underpinned these strategies. The experience offered an opportunity to assess the contribution of implementation research (IR) to VL elimination in Nepal. Desk review and a stakeholder workshop was conducted to analyse the relationship between key research outputs, major strategic decisions in the national VL elimination programme, and annual number of reported new cases over time between 2005 and 2023. The results indicated that the key decisions across the strategic elements, throughout the course of the elimination programme (such as on the most appropriate tools for diganostics and treatment, and on best strategies for case finding and vector management), were IR informed. IR itself responded dynamically to changes that resulted from interventions, addressing new questions that emerged from the field. Close collaboration between researchers, programme managers, and implementers in priority setting, design, conduct, and review of studies facilitated uptake of evidence into policy and programmatic activities. VL case numbers in Nepal are now reduced by 90% compared to 2005. Although direct attribution of disease decline to research outputs is difficult to establish, the Nepal experience demonstrates that IR can be a critical enabler for disease elimination. The lessons can potentially inform IR strategies in other countries with diseases targeted for elimination.

## Author summary

Investigators supported by TDR, WHO, and other partners engaged with national stakeholders (policy makers, implementers, clinicians, public health workers, and others) in the development and validation of strategies for elimination of visceral leishmaniasis (VL) in Nepal in the context of the regional Kala Azar Elimination Programme. Linkage and regular consultations with the national programme guided the research focus and facilitated continued uptake of results into policy and implementation of evidence-based interventions. Knowledge from implementation research led to public health programme decisions that facilitated VL elimination in Nepal.

## Background

Visceral leishmaniasis (VL), also known as kala-azar (KA), is a fatal parasitic disease, if untreated. It is transmitted by sandflies, with anthroponotic transmission (in the Indian subcontinent), mostly affecting socially deprived populations [1–4]. Worldwide, an estimated 50,000 to 90,000 new VL cases occur annually with more than 90% of cases reported from just 10 countries, including Bangladesh, India, and Nepal [5–11].

Nepal signed a Memorandum of Understanding (MoU) with Bangladesh and India in 2005 to eliminate VL as a public health problem from the region by 2015 [12]. By 2021, the number of reported VL cases in these countries had declined by over 95% compared to 2007 [13]. The target of the MoU was to achieve disease elimination (new case rate < 1/10,000 population at the district level) by 2015, through 3 phases of preparatory (2005 to 2008), attack (2008 to 2015), and consolidation (2015 onwards). The 5 strategic elements for elimination were early case diagnosis and complete treatment, integrated vector management (IVM), effective disease surveillance, social mobilization and partnership, and operational research.

The UNICEF/UNDP/World Bank/WHO Special Programme for Research and Training in Tropical Diseases (TDR), World Health Organization (WHO), and several other partners have coordinated and financed implementation research (IR) in support of the VL elimination initiative in the Indian subcontinent before and throughout the course of the regional elimination programme. Available data on IR in the Indian subcontinent with a particular focus on Nepal, which covered all strategic elements and sustained throughout the course of the programme, may provide useful insight from the national perspective on the contribution of IR to achieving disease elimination targets.

The objective of the review is to describe and analyse key characteristics, contributions, milestones, and impact of IR on the elimination of VL as a public health problem in Nepal.

## Methodology

A desk review of relevant VL publications, documents on VL elimination, and strategies and operational plans of Nepal or relevant to Nepal (from internal and external sources) was conducted.

Expert consultations were held with key stakeholders including national VL programme managers, VL treating clinicians, entomologists, and researchers to collect information on implementation or operational research activities and alignment with the programme and IR needs to achieve sustainability of elimination. The consultations included national program officers and the WHO country office.

A 5-day workshop (23 to 27 September 2021) was organized with academic institutions, national programme managers, and development partners. The workshop discussed on the role and contributions of IR to the elimination effort so far and identified the research and programmatic priorities to achieve and maintain VL elimination going forward. The discussion points and recommendations of the workshop have been provided in findings and discussion sections below.

### Findings

IR on VL in Nepal covered all the strategic elements of the Kala-Azar Elimination Programme. TDR-supported country-led IR established the burden of VL, characterized population health-seeking behaviour, investigated feasibility of new interventions, identified barriers to early diagnosis and treatment, developed strategies for cost-effective active case detection (ACD), demonstrated effective and long-lasting vector control interventions, explored environmental determinants (e.g., housing as a risk factor for VL transmission), and examined the role of frontline health workers. Based on the findings from these studies and other similar research, which also fed into regional consensus, the national VL elimination program developed strategies, guidelines, and implementation plans for the attack phase of VL elimination in line with the recommendations of the WHO regional technical advisory group (RTAG), within the context of the WHO-supported regional VL elimination strategy [14,15].

Key decisions underpinned by sustained IR that directly impacted on reduced case burden in Nepal included the following: (i) rK39-based rapid test employed as a confirmatory test for VL as of 2005, following extensive field validation; (ii) miltefosine (MIL) replaced sodium stibogluconate (SSG) as a first line of treatment in 2008 to 2014; (iii) liposomal amphotericin B (L-AmB) replaced MIL in 2015 in response to increased treatment failures and relapse rates; (iv) combination therapy was introduced in Nepal’s national treatment protocol in 2014; (v) active case detection (ACD) was incorporated into the national VL elimination protocol; and (vi) integrated vector management (IVM) was recognized as an important element in the elimination efforts.

### VL trends in Nepal 2002–2020

**Fig 1:**
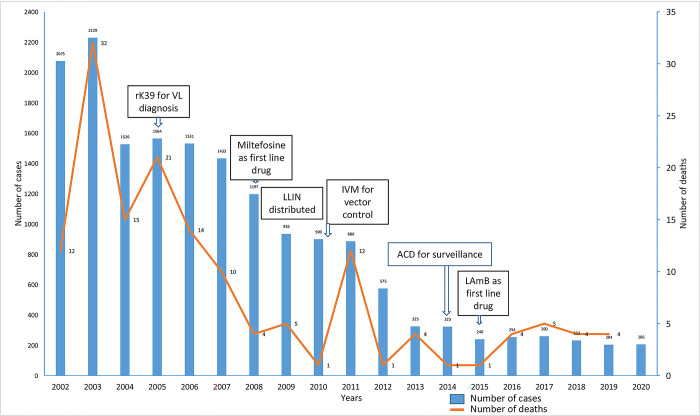
Trend of VL cases and deaths and major interventions in Nepal, 2002–2020.

In 2003, Nepal witnessed the highest case load of VL, although there were many outbreaks of VL in the past [16]. In the same year, the rotation of synthetic pyrethroids for vector management was introduced. The overall trend of VL cases decreased significantly thereafter. The number of cases and deaths has been steadily falling since the VL elimination initiative started in 2005 (Fig 1). In 2008, MIL was introduced as oral monotherapy. The number of cases decreased further in comparison to previous years. This could be due to the convenience of the oral medication influencing patient compliance to treatment. Around the same period in 2009, long-lasting insecticide-treated nets (LLINs) were introduced in addition to indoor residual spraying (IRS), two measures that helped decrease the man–vector contact and, thus, the VL burden in the country. In addition, ACD was introduced into the VL elimination program in 2014 after a series of large intervention studies to identify the most cost-effective way of ACD. Overall, the number of VL cases has decreased by 90% in 2020 as compared to 2002, which is most likely a result of the synergistic impact of all interventions of the VL elimination program.

### Diagnostic tests for VL

The direct agglutination test (DAT) had been in use as a diagnostic tool for VL since the early 90s following its assessment through TDR-supported research, which had confirmed it to be specific and sensitive and relatively acceptable for field laboratory settings [17,18]. In subsequent years, rK39-based rapid test was found to be more cost-effective and simpler to use in field settings [19–24]. The heterogeneity in test results of rK39 could be due to geographic region, commercial brand, disease prevalence, sample size in the study, type of reference standard, and risk of bias during testing [25]. The sensitivity of rK39 is 97% and specificity is 90.2% in Indian subcontinent, and because of ease of use [25], it was recommended for diagnosis of VL in Indian subcontinent. rK39 was introduced as a diagnostic tool in the national VL elimination program in Nepal in 2005. The key studies on VL diagnostics have been given in Table 1.

**Table 1:** Research on diagnostic tests for VL.

Author Year	Funder	Study Design	Study Site(s) and Year	Results	Conclusions
Joshi et al., 1999 [17]	TDR	Diagnostic evaluation of DAT before its field application	Siraha, Kathmandu; 1993–1994	Sensitivity: 100%; Specificity: 99.2% Positive predictive value: 100%; Negative predictive value: 99.2%	DAT is simple, rapid, reliable, economic, safe, and adaptable.
Bern et al., 2000 [19]	USAID	Diagnostic evaluation (rK39 and DAT)	Dhanusha, Mahottari Nepal; 1998	rK39 dipstick test Sensitivity: 100% Specificity: 100% DAT sensitivity: 100% Specificity: 93%	rK39 is more advantageous, simple to perform and interpret in the field setting.
Chappuis et al., 2003 [20]	TDR	Diagnostic evaluation (rK39, DAT) study 184 VL patients	BP Koirala Institute of Health Sciences, Dharan; 1999–2000	rK39 ICT sensitivity: 97%, specificity: 71%; DAT sensitivity: 99%, specificity: 82%	rK39 ICT equates well with DAT and is easy to use in field settings.
Boelaert et al., 2004 [21]	TDR	Diagnostic (rK39 ICT, FGT, IFAT, DAT) evaluation study	BP Koirala Institute of Health Sciences, Dharan; 2000–2002	rK39 ICT sensitivity: 87.4%, specificity: 93.1%; FGT sensitivity: 39.9%; IFAT sensitivity: 28.4%; DAT sensitivity: 95.1%	DAT and rK39 ICT can replace VL diagnosis via bone marrow or splenic aspiration.
Chappuis et al., 2006 [22]	TDR	Diagnostic (rK39 ICT, FGT, KAtex) evaluation study	Rangeli Hospital, Morang; 2001–2002	rK39 sensitivity: 89%, specificity: 90%; FGT sensitivity: 52%; KAtex sensitivity: 57%; Reproducibility higher for rK39 (k = 0.87) compared to FGT and KAtex	rK39 meets most of the criteria of ASSURED.
Das et al., 2007 [23]	BP Koirala Institute of Health Sciences	Evaluation of diagnostic tool for PKDL and VL patients	BP Koirala Institute of Health Sciences, Dharan; 2005	DAT sensitivity: 100% specificity: 93% rK39 sensitivity: 96% specificity: 100%	rK39 is cost effective, easy to use and store in field condition comparatively.
Boelaert et al., 2007 [25]	Institute of Tropical Medicine, Antwerp	Review of considerations for evaluation of diagnostic tests (test for case detection, cure, relapse, surveillance, drug resistance, certification of elimination)	Review	Lower specificity (71%) in Nepal in early prototype; higher specificity in later generation of InBios ICT and DiaMed ICT	Standardized methodology for the evaluation of RDT is required to prevent the substandarization of product being used.
Boelaert et al., 2008 [26]	TDR	Diagnostic (rK39 ICT, DAT-FD, KAtex) evaluation study 1,150 VL patients	Bangladesh, India, Nepal, Ethiopia, Kenya, Sudan; 2006–2007	DAT-FD: sensitivity: 98.5%; specificity: 95.4%. rK39: sensitivity: 96.5%; specificity: 90.9%. KAtex: sensitivity: 35.8%; specificity: 97.8%	DAT-FD, rK39 ICT recommended for Indian subcontinent.
Deborggraeve et al., 2008 [27]	European Communities	Diagnostic accuracy of PCR in 173 VL patients	BP Koirala Institute of Health Sciences, Dharan; 2000–2002	Sensitivity: 92.1% in blood, 92.9% in bone marrow Specificity: 99.64%	High sensitivity and specificity but should be used with standardized clinical VL case definition.
Boelaert et al., 2014 [28]	Institute of Tropical Medicine, Antwerp	Review of accuracy of rapid diagnostic test	Review	rK39 ICT sensitivity: 97%, specificity: 90.2%; Latex agglutination test sensitivity: 50.8%, specificity: 95.3%	rK39 ICT recommended in patients with febrile splenomegaly and no past VL history.
Singh and Sundar, 2015 [29]	National Institute of Allergy and Infectious Disease	Review of current diagnostic test	Review	rK39 showed high diagnostic accuracy. DAT showed satisfactory results.	Standardized clinical case definition should also be used while making diagnosis for VL.
Shrestha et al., 2019 [30]	Nepal Academy of Science and Technology	Confirmation of *Leishmania* species via molecular approach in VL	Sukraraj Tropical and Infectious Disease Hospital Kathmandu; 2015–2016	PCR: Positive: 2 Negative: 4. 2 positive PCRs were also positive for bone marrow aspiration.	Molecular tests should also be incorporated for better understanding of the origin and spread of these strains.
Cloots et al., 2020 [31]	The Directorate-General for Development Cooperation of Belgium	Assessment of DAT as a marker of infection for future surveillance.	Sunsari, Saptari, Morang; 2016	DAT prevalence for children <10 years: (2006): 4.1%, (2016): 0.5%	DAT could be a useful tool for monitoring the transmission of *L*. *donovani*.

DAT, direct agglutination test; FD, freeze dried; FGT, formol gel test; ICT, immunochromatographic test; IFAT, immunofluorescence antibody test; PCR, polymerase chain reaction; PKDL, post kala-azar dermal leishmaniasis; RDT, rapid diagnostic test; TDR, UNICEF/UNDP/World Bank/WHO Special Programme for Research and Training in Tropical Diseases; VL, visceral leishmaniasis.

### Treatment regimens for visceral leishmaniasis

SSG was first introduced in Nepal in 1982 and became a first-line choice for treatment of VL in 1994. The study conducted by Karki and colleagues [32] on SSG revealed satisfactory results demonstrating better efficacy in those treated for a longer period. However, SSG showed increasing failure rates of 10% to 30% in Nepal [33] and 65% in Bihar [34] at the turn of the century. Amphotericin B was introduced for the treatment of VL in 1994. Subsequently, a TDR-supported Phase IV trial of MIL [35] showed that it was a safe and effective oral drug for treatment of VL. Oral monotherapy with MIL was introduced in 2008 as a first-line drug for VL in the absence of contraindications, until revised in 2014. Following the introduction of MIL as a first-line regimen, TDR supported another Phase IV trial conducted by Banjara and colleagues [36] in Saptari district. Although MIL was relatively safer and easier to administer than SSG [37], it is potentially teratogenic and, hence, contraindicated in pregnancy and during breastfeeding. The study by Rijal and colleagues [38] reported a 10% to 20% relapse rate after treatment with MIL. Banjara and colleagues [36] and Uranw and colleagues [39] showed that poor adherence and insufficient dose were associated with the occurrence of relapses. On the other hand, MIL is, in some case, a useful drug as a rescue treatment for patients with VL relapse [40].

TDR-supported trials conducted in the Indian subcontinent showed that combined therapies had a favourable outcome for VL treatment [41–44]. These studies revealed that combined therapy reduces the dose requirements without compromising the cure rate. The national treatment protocol of 2014 introduced combined therapy as a second-line treatment for VL. The combination regimen consists of L-AmB (5 mg/kg, single infusion) plus 7 days MIL (50 mg 2 times a day or, alternatively, L-AmB (5 mg/kg, single infusion) plus 10 days paromomycin (PM) (11 mg/kg base) or MIL plus PM for 10 days. Combined therapy has replaced amphotericin B monotherapy as a second-line treatment shifting it to third-line treatment in the national guideline of 2019.

The TDR-supported dose finding studies conducted in India showed that L-AmB was safe, effective, and favorable for the treatment of VL with minimal toxicity [41,45]. In 2014, L-AmB was introduced into the treatment protocol for VL and is, since 2015, the recommended first-line drug in the national guideline for VL elimination.

Table 2 presents the key studies on drugs for VL treatment.

**Table 2:** Research on VL treatment (drugs).

Drugs for VL Treatment	Author	Funder	Study Design	Study Site (s) and Year	Results	Conclusions
SSG	Karki et al., 1998 [32]	BP Koirala Institute of Health Sciences	Evaluation of SSG efficacy at a dose of 20 mg/kg/day for 20 days and 30 days.	BP Koirala Institute of Health Sciences; 1994–1997	For 20 days efficacy: 77.78% For 30 days efficacy: 92.59%	Longer period (30 days) treatment is more effective with higher cure rate and minimum side effects.
SSG	Rijal et al., 2003 [33]	Geneva University	Evaluation of SSG efficacy at a dose of 20 mg/kg/day for 30 days 110 VL patients	BP Koirala Institute of Health Sciences; 1999–2001	Cure: 90% Failure: 10%	SSG efficacy is satisfactory except for those living near antimony-resistant VL areas of India.
SSG	Rijal et al., 2010 [46]	European Union	Evaluating the risk factors for the therapeutic failure of SSG	BP Koirala Institute of Health Sciences; 2001–2003	Cure: 90.5% Failure: 9.5%	Cure rate, comparing from the view of intention-to-treat is much lower (77.3%).
MIL	Bhattacharya et al., 2007 [35]	TDR	Evaluation of the feasibility of VL treatment	Bihar, India; 2004–2006	Treatment compliance 95.5% and 85.8% for 28 and after 6 months	Oral MIL is safe and effective except teratogenicity
MIL	Banjara et al., 2012 [36]	TDR	Evaluation of clinical management of VL after intervention	Saptari Nepal; 2010	Use of MIL was the same as amphotericin B.	Counselling by physicians and availability of drugs plays a significant role in prescription behaviour.
MIL	Uranw et al., 2013 [39]	Belgian Federal Cooperation	Evaluation of adherence to MIL	BPKIHS, Lahan Hospital, Mahottari Hospital; 2010–2011	Adherence: 83%	Poor adherence was associated with relapse. Effective counselling, understanding of side effects, action of MIL along with home visit can prevent poor adherence.
MIL	Rijal et al., 2013 [38]	European Commission	Evaluation of MIL clinical outcome upto 12 months	BPKIHS, Dharan; 2008–2011	Cure: 95.8% Relapse at 6 months: 10.8% Relapse at 12 months: 20%	Effectiveness of MIL has reduced with increasing relapse
MIL	Dorlo et al., 2014 [47]	European Union	Evaluate the relationship between MIL drug exposure and treatment failure with VL.	BPKIHS, Dharan; 2010–2011	Children (<12 years) are risk factors for treatment failure.	Sufficient dose of MIL is required to achieve successful VL treatment.
L-AmB	Thakur et al., 1996 [41]	TDR	Evaluation of 3 regimens of L-AmB (total doses: 14 mg/kg, 10 mg/kg, and 6 mg/kg) for VL treatment.	Bihar, India; 1994–1995	Clinically cured in all patients by 24th day. No relapse within 12 months follow-up.	L-AmB is safe and highly efficacious for VL treatment with minimal side effect and toxicity.
L-AmB	Sundar et al., 2003 [45]	Gilead Science	Evaluation of single-dose L-AmB (7.5 mg/kg) in India.	Bihar, India; 2001	Cure: 90% Failure: 10%	Single-dose L-AmB treatment is safe and effective and can be used for mass treatment of VL.
L-AmB and amphotericin B	Sundar et al., 2004 [48]	TDR	Evalution of efficacy of L-AmB (2 mg/kg for 5 days) vs amphotericin B (1 mg/kg on alternate days for 30 days).	Bihar, India; 2001	Final cure rates were similar in both treatment.	L-AmB is safe as well as better tolerated.
L-AmB	Sinha et al., 2010 [49]	The Medecins Sans Frontieres	Evaluation of L-AmB’s effectiveness and safety	Bihar, India; 2007–2008	Intention-to-treat: 98.8%; per protocol: 99.6%; Intention-to-treat worse-case scenario: 81.3%	Safe and effective but reduced dose and combination with partner drugs can also be effective.
L-AmB and amphotericin B	Sundar et al., 2010 [43]	TDR	Evaluation of single-dose L-AmB in comparison to conventional amphotericin B deoxycholate	Bihar, India; 2008–2009	Cure at 1 month: L-AmB therapy: 100%, Conventional therapy: 98%	Single infusion L-AmB is not inferior to conventional therapy.
PM	Jha et al., 1998 [50]	TDR	Evaluation of PM’s efficacy and tolerability	Bihar, India; 1993–1995	Cure for PM was 77%; 93% and 97%	A 21-day course of PM at the dose of 16 or 20 mg/kg/day can replace SSG as a first line of treatment.
PM	Sundar et al., 2007 [51]	One World Health; TDR	Evaluation safety and efficacy of PM in comparison to amphotericin B	Bihar, India; 2003–2004	Final cure rate at 30 days: PM: 94.6%; Amphotericin B: 98.8%	PM is safe and efficacious and noninferior to amphotericin B.
PM	Sinha et al., 2011 [52]	One World Health	Evaluation of PM’s safety and efficacy	Bihar, India; 2009–2010	Dose:11 mg/kg Initial cure at 21 days: 99.6%; Final cure after 6 months: 94.2%; Adverse effect: 5%;	PM is safe and efficacious to use in an outpatient setting.
Combined therapy (PM + SSG)	Thakur et al., 1996 [53]	TDR	Evaluation of safety and efficacy of PM	Bihar, India; 1993–1994	Cure: (PM 12 mg/kg/day + SSG 20 mg/kg/day): 88%; (PM 6 mg/kg/day + SSG 20 mg/kg/day): 69%	Combined therapy with PM at 12 mg/kg/day for 20 days is more effective and can be considered as a safe replacement for SSG alone for 40 days.
Combined therapy (PM + SSG)	Thakur et al., 2000 [42]	TDR	Evaluation of the safety and efficacy of PM	Bihar, India; 1996	Final cure: PM 12 + SSG: 92.3%; PM 18 + SSG: 93.8% SSG alone: 53.1%	Combined dose of PM at 12 or 18 mg/kg with standard dose for SSG for 21 days was more effective than SSG alone.
Combined therapy (L-AmB + MIL)	Sundar et al., 2011 [44]	TDR	Evaluation of safety and efficacy of L-AmB (5 mg/kg) plus MIL (2.5 mg/kg/day for 14 days). 135 VL patients.	Bihar, India; 2009	Intend to treat cure rate at 6 months: 91.9%; Per protocol cure rate: 97.6% (124 of 127 evaluable patients). Side effects were fever, rigors, and back pain due to L-AmB; gastrointestinal side effects by MIL.	Combination therapy has good efficacy, tolerance, and feasibility of administration without compromising the cure rate.

L-AmB, liposomal amphotericin B; MIL, miltefosine; PM, paromomycin; SSG, sodium stibogluconate; TDR, UNICEF/UNDP/World Bank/WHO Special Programme for Research and Training in Tropical Diseases; VL, visceral leishmaniasis.

### VL vector control

Joshi and colleagues (2008), Joshi and colleagues (2009), and Das and colleagues (2010) [54,55,56] in their TDR-funded study found that integrated vector management (IVM) can play a vital role in the VL elimination program. This was subsequently introduced into the 2010 national guidelines for VL elimination. TDR-funded studies have found that indoor residual spraying (IRS), insecticide-treated nets (ITNs), and insecticidal wall painting (IWP) are effective ways to control sandflies in Nepal [57,58]. IRS with DDT, Malathion, and Lambda Cyhalothrin (ICON) had been in use from 1992 until 2002 [59,60]. From 2003 onwards, the rotational substitution of synthetic pyrethroids was introduced in order to avoid vector resistance. Alpha cypermethrin was used in 2003 and 2012, while Lambda Cyhalothrin was reintroduced in 2007. A Lambda cyhalothrin study funded by TDR showed 97% mean vector mortality and a beneficial impact of IRS for up to 4 weeks after insecticide spray [61]. However, the substandard performance and management of the spraying activity increased the risk of insecticide resistance [60,62,63]. The studies by Joshi and colleagues (2009), Picado and colleagues (2009), and Picado and colleagues (2010) [55,64,65] did not show a significant reduction of VL incidence after the use of LLINs, but the study by Das and colleagues (2010), Mondal and colleagues (2016), and Das and colleagues (2012) [56,58,66] found LLINs to be a favorable alternative to IRS. Key studies on vector control are shown in Table 3.

**Table 3:** IR on VL vector control.

Intervention	Author	Funder	Study Design	Study Site (s) and Year	Results	Conclusions
IRS	Joshi et al., 2003 [67]	TDR	Analyse the effectiveness of the IRS.	Siraha, Nepal; 2001–2002	Disease incidence was found to be same before and after insecticide spray	The resistance was developed due to haphazard use of insecticides.
IRS, LLIN, IVM	Joshi et al., 2009 [55]	TDR	Evaluate the efficacy of different intervention VL vector management.	Fulbaria Bangladesh, Vaishali and Muzzaffarpur India, Sarlahi, Sunsari, and Morang Nepal; 2006	IRS showed significant reduction in sandfly densities. LLINs didn’t have a significant negative effect on the density of sandflies. IVM using lime plastering had significant sandfly density.	Vector control provides support to the VL elimination program.
IRS, LLIN, IVM	Das et al., 2010 [56]	TDR	Comparative study of KA vector control measures in Nepal	Sunsari, Morang Nepal; 2006	LLIN and pyrethroid-based insecticides effectively reduced the vector density IVM also appeared to reduce vector density.	IRS and LLINs, with proper supervision, were found to be the most effective measure to control vector density. IVM can be considered to be integrated with other interventions.
IRS	Dinesh et al., 2010 [60]	European Union	Analyse the sensitivity of *P*. *argentipes* to 4% DDT and 0.05% deltamethrin	Bihar India and South-eastern Nepal; 2008–2009	DDT mortalities varied from 62% to >90%. Deltamethrin had 96%-99% mortality.	DDT resistance was confirmed in villages on the border with Bihar. Susceptibility towards deltamethrin is high.
IRS	Chowdhury et al., 2011 [61]	TDR	Performance and effectiveness of lambda-cyhalothrin as IRS.	Vaishali India, Sarlahi and Sunsari Nepal; 2008–2009	Mean mortality of *P*. *argentipes* when exposed to deltamethrin was 97%.	Substandard spraying may contribute to development of insecticide resistance.
LLIN	Picado et al., 2010 [65]	European Union	Assessment of PermaNet LLIN in the reducing VL incidence	Muzaffarpur India, Saptari, Sunsari, Morang Nepal; 2006	Risk of seroconversion: 3.1% Risk of VL after LLIN intervention was reduced by 10%	The results after the use of LLIN were not significant.
LLIN	Banjara et al., 2015 [68]	TDR	Assessment of bednet impregnation with KOTAB 123 for vector control.	12 VL endemic villages of Bangladesh, India, Nepal; 2013–2014	Untreated bednet: 89.3%; Regular bednet use: 67.7%; Sandfly reduction: At 2 weeks: 32.6%; At 4 weeks: 12.5%	No significant change after insecticide impregnation
DWL (IVM)	Huda et al., 2016 [69]	TDR	Assessment of DWL and RWSC for vector control.	3 endemic villages each in Bangladesh and India and 3 endemic clusters in Nepal; 2014	Both interventions had a significant decrease in sandfly density.	DWL has the potential to be a long-term vector control tool. Due to its cost-efficacy over FWSC, RWSC can be considered for the VL elimination program
IRS LLIN IWP	Banjara et al., 2019 [57]	TDR	Assessment of vector control methods.	Saptari Nepal; 2015	12-month sandfly mortality and effectiveness: IRS with deltamethrin: 23%, 1 month Bednet impregnated with KOTAB123: 26%, 1 month Wall painting with Inesfly 5AIGRNG: 80%, 12 months	Wall painting with insecticidal paint can be considered as an alternative and sustainable strategy in the VL post elimination program.
IVM	Younis et al., 2020 [70]	TDR	Assessing the role of housing conditions and its environment in VL vector occurrence and transmission.	Morang, Saptari, Palpa Nepal; 2018	AOR: Bamboo wall: 2.9; Walls made of leaves/branches: 3; Cracks in bedroom walls: 2.9; Sacks near sleeping area: 19.2; Kadam trees: 12.7; Open ground outdoor toilet: 9.3; Moisture in outdoor toilet shed: 18.09; Open land: 36.8; Moisture inside animal shed: 6.9; Surrounding animals/animal waste: 3.5	Elimination and educational programs should enforce awareness on housing improvement.

AOR, adjusted odd ratio; DWL, durable wall lining; FWSC, full wall surface coverage; IR, implementation research; IRS, indoor residual spraying; IVM, integrated vector management; IWP, insecticidal wall painting; KA, kala-azar; LLIN, long-lasting insecticide-treated net; RWSC, reduced wall surface coverage; TDR, UNICEF/UNDP/World Bank/WHO Special Programme for Research and Training in Tropical Diseases; VL, visceral leishmaniasis.

### VL case detection and surveillance

Various TDR-funded studies have explored different models of ACD reaching a similar conclusion that ACD is a cost-effective approach and appropriate for the elimination program [16,71–74]. Singh and colleagues [72], in their TDR-funded study, assessed different ACD strategies in order to identify the most cost-effective approach and periodicity of ACD. They showed that the “camp approach” (periodic fever camps with VL screening) is recommended for high VL endemic areas, the “index approach” (perifocal screening around a VL case) for high to moderate VL areas, and the “incentive approach” (payment of community health workers for each case detected) in low endemic areas. Banjara and colleagues [68] also emphasized the needed reinforcement of community health workers. One of the early TDR-funded studies conducted in rural Nepal had already highlighted the need for optimal engagement of the local health workers to encourage villagers to actively participate in the VL control program [75]. In the recent TDR study by Lim and colleagues [76], it was found that the delay in case reporting from the first symptoms to start of appropriate treatment was 68 days in VL program districts and 83 days in non-program (new foci) districts. Similarly, the diagnostic delay for program and non-program districts was 38 and 36 days, respectively. In order to facilitate a responsive intervention, delivery of timely and reliable information towards awareness of VL and its prompt treatment is essential. Table 4 summarizes key studies on surveillance of VL.

**Table 4:** IR on VL surveillance.

Surveillance Strategy	Author	Funder	Study Design	Study Site (s) and Year	Results	Conclusions
ACD: House screening	Mondal et al., 2009 [71]	TDR	Evaluate the health-seeking behaviour for VL to provide baseline information for VL elimination program and explore potential for ACD.	Rajshahi Bangladesh, Vaishali and Muzaffarpur India, Mahottari Nepal; 2006–2007	ACD: 37.5%; VL knowledge: 82%; Public sectors were preferred (45%) over private sectors (11%).	ACD via house screening seems feasible; however, other models of ACD should be explored along with their operational cost.
ACD: index case-based; PCD	Hirve et al., 2010 [72]	TDR	Assess the effectiveness and feasibility of ACD and PCD in VL elimination strategy.	Mymensingh Bangladesh, Saran, Muzaffarpur India, Saptari, Sunsari, Morang Nepal; 2008	7 new VL cases. Total direct cost of ACD: USD1,836. ACD is cost-effective when disease burden is high.	ACD can be a cost-effective approach supporting PCD. It decreases delay in diagnosis and treatment.
ACD: -Camp -Blanket approach -Index case approach -Incentive approach	Singh et al., 2011 [73]	TDR	Assess the feasibility, cost, and effectiveness of ACD strategies. 2 rounds 6 months apart.	Mymensingh Bangladesh, Saran, Muzaffarpur India, Sarlahi Nepal; 2009	Round 1 Camp: New VL cases: 5, Sensitivity: 100%; Blanket: New VL case: 5; Index: New VL cases: 0, Sensitivity: 0%; Incentive: New VL cases: 4, Sensitivity: 100%; Cost per camp (USD): 195; Round 2 Camp: New VL cases: 3, Sensitivity: 100%; Blanket: New VL case: 3, Index: New VL cases: 0, Sensitivity: 0%.	Adapting camp approach in high VL endemic area, index approach in high to moderate VL endemic area, and incentive approach in low VL endemic area are recommended.
ACD: -Camp -Index case-based	Huda et al., 2012 [74]	TDR	Assess the feasibility and performance of ACD strategies in national program. 2 Camps and 45 Index case searches at 37 VDCs in endemic areas.	Bangladesh: 4 Upazillas, India: 9 PHCs, Nepal: 37 VDCs; 2010–2011	Camp strategy submitted 3 new VL cases. One camp lacked adequate supply of rK39 for testing. No new case from index case search strategy. Cost of detecting the new VL was USD 199.	ACD can be adapted in national program; however, its challenges has to be overcome via appropriate training, planning, and strengthening referral service.
ACD -Combined camp	Banjara et al., 2015 [68]	TDR	Assess the feasibility and result of ACD via combined camp strategies.	12 VL endemic villages of Bangladesh, India, Nepal; 2013–2014	New VL case: 1; Cost per combined camp: USD 125.15; rK39, drugs, and other supplies for VL treatment were available in district health facilities.	Combined fever camps seem to be feasible; however, the cost for the health services need further rumination. Cost-efficacy can be achieved when combined camps are part of routine operation.
Passive surveillance	Boettcher et al., 2015 [77]	TDR	Assess the passive surveillance and analyse the duration required for reporting of VL cases from district to central health authorities.	12 districts of Nepal, 9 districts of Bihar India; 2012	Seeking health care: 30 days; Receiving VL diagnosis: 25 days; Receiving treatment: 3 days; No. of consultation: 1.4 Reporting of VL case: 77 days	Implementation of the electronic VL reporting system and close link to HMIS by the central level can be beneficial.
Passive surveillance	Lim et al., 2019 [76]	TDR	Assess the surveillance and reporting system requiring strengthening.	Sarlahi, Saptari, Sunsari, Morang (program districts), Palpa, Okhaldhunga, Bhojpur, Nuwakot (non-program districts); 2016	Delay reporting: Program district: 68.5 days, Non-Program district: 83 days; Diagnostic Delay: Program district: 38.5 days, Non-Program district: 36 days; Reporting Delay: Program district: 45 days, Non-Program district: 36 days.	Awareness on VL along with ensuring prompt treatment, timely and reliable information is needed to facilitate a responsive system of interventions.
ACD (incentive approach)	Omer et al., 2020 [78]	PHIDReC	Analyze the existential and potential role of FCHV in VL elimination	Saptari, Morang, Palpa Nepal; 2018	FCHVs in Terai region were more aware of VL than those of hilly region. Communication gaps between FCHV and health officials were present. Knowledge of VL was better in households with a history of KA than those without.	FCHVs play an important role in the VL elimination program as they are living in the community and their participation can make the program sustainable and efficient. Formal training.
Passive surveillance	Cloots et al., 2020 [31]	Development Cooperation of Belgium	Assessment of *L*. *donovani* transmission since the launch of VL elimination initiative.	Sunsari, Saptari, Morang; 2016	Seroprevalence was lower in 2016 as compared to 2006. Adjusted risk ratio of 2016 in comparison to 2006 for seropositive: 0.44	Current surveillance is based on the monitoring of disease incidence; nevertheless, monitoring of infection would permit more accurate surveillance.

ACD, active case detection; FCHV, female community health volunteer; HMIS, health management information system; IR, implementation research; KA, kala-azar; PCD, passive case detection; PHC, Primary Health Care Centers; TDR, UNICEF/UNDP/World Bank/WHO Special Programme for Research and Training in Tropical Diseases; VDC, Village Development Committee; VL, visceral leishmaniasis.

## Discussion

Nepal eliminated VL as a public health problem at the district level in 2014 and has sustained the status for 3 years [79]. Evidence shows that transmission of *Leishmania donovani* in Nepal has decreased significantly during the elimination programme [31]. Despite the recent emergence of new foci in some hilly districts requiring further exploration and intervention, the KA elimination programme in Nepal is a success story [31].This achievement is obviously an outcome of converging inputs from multiple health system blocks driving effective information systems, access to essential medicines, financing, and leadership/governance [80], including strong political commitment internally, as well as the advantages accruing from a regional approach (with successful reduction of disease burden during the same period in Bihar, India, bordering with Nepal).

Key drivers of success that are frequently cited are interventions and tools such as rapid diagnosis with rK39 RDT, active case finding, treatment with L-AmB, and IRS [14]. The research that underpins these tools and their implementation receives less attention. However, effective IR would be expected to have contributed to strategic decisions in the choice and implementation of the interventions and guided their implementation across these strategic elements. The impact of IR on disease burden is difficult to measure because it acts through downstream drivers limiting direct attribution. Long-term interventions such as the VL elimination programme where IR is integrated into the strategy offer opportunities to explore the contribution of IR to the achievement of public health targets.

One of the early research projects supported by TDR described peoples’ knowledge, attitude, and practice regarding VL in rural Nepal. The data obtained from this study helped in planning and evaluating VL-related control activities.

The VL elimination program benefitted from successive innovations, which facilitated early diagnosis (field-friendly rapid confirmatory test), effective treatment (MIL, L-AmB), and ACD based on VL burden (camp, household, index-based) and effective vector control (LLINs, IRS). These measures were introduced at various stages in the course of the elimination programme following policy decisions at the national and regional levels based on evidence and the recommendations of the RTAG of WHO. The success of these interventions is evident in the rapid decline of reported cases and death over the years (Fig 1). Because IR lies behind these policy decisions, one can safely conclude that IR has contributed to reduction in disease burden, albeit indirectly through evidence uptake into policy.

### Lessons and perspectives

The VL elimination program in the Indian subcontinent implemented a package of clearly defined evidence-based interventions and an effective performance management process. It could sustain partnerships that contributed in different ways and communicated well with the program. It benefitted from a strong political commitment. An important component among these key areas for public health program success that the program enjoyed was a research platform to develop the evidence base for action [81].

In addition to scientific publications, standard operating procedures (SOPs), policy briefs, monitoring and evaluation (M&E) handbooks, and other operational documents were developed by researchers and programme staff in collaboration with WHO, TDR, and national partners. These have largely been adopted by the national authorities. The input, together with findings generated through operational research supported by other development partners in these countries, has shaped the RTAG’s recommendations and helped advance the VL elimination effort in Nepal (as in the other countries).

Every year, TDR organizes joint meetings of program managers, country researchers, TDR, WHO/NTD, WHO-SEARO, RTAG, DNDi, McGill University, and other partners to update participants on the progress and challenges of the program as well as to share new IR findings, identify research priorities, and draft the proposals for the next research phase. Country researchers have participated in the development of national and regional strategic plans and programs. RTAG meeting recommendations were translated into the strategies for VL elimination in the country (Table 5).

**Table 5:** Major strategic changes in the VL control/elimination program in Nepal.

Particulars	Introduced in National Elimination Program
***Diagnostic tool***	
DAT	1994
rK39	2005
***Treatment***	
L-AmB (monotherapy)	2014 introduced in national treatment protocol, in 2015 as first-line therapy
MIL (oral monotherapy)	2008 as first-line regimen 2019 as fourth-line regimen
Amphotericin B deoxycholate (monotherapy)	2019 as third-line regimen
PM (monotherapy)	2019 as fourth-line regimen
L-AmB 5 mg/kg (single) + MIL 50 mg BD (7 days) OR L-AmB 5 mg/kg (single) + PM 11 mg/kg (10 days), OR MIL + PM (10 days) (Combination therapy)	2014 introduced in national treatment protocol as second line of treatment
SSG (monotherapy)	1994 as first-line regimen
***Vector control***	
IRS with Lambda Cyhalothrin (ICON), DDT, Malathion	1992 till 2002
IRS with Alpha Cypermethrin	2003
IRS with Lambda Cyhalothrin (ICON)	Reintroduced in 2007
IRS with Alpha-Cypermethrin	2012
IRS with Deltamethrin	2014
IRS with Lamda Cyhalothrin	2017 till 2020
ITNs distribution	2009
IVM	2010
***Surveillance***	
ACD	2014
SOP for ACD	2014

ACD, active case detection; DAT, direct agglutination test; IRS, indoor residual spraying; ITN, insecticide-treated net; IVM, integrated vector management; L-AmB, liposomal amphotericin B; MIL, miltefosine; PM, paromomycin; SOP, standard operating procedure; SSG, sodium stibogluconate; VL, visceral leishmaniasis.

The lessons learned and the consistent documentation of the regional achievements could serve as reference for VL elimination in Africa. For Nepal to sustain progress towards the elimination goal, more efficient and effective methods for ACD and vector management, which respond to the changing epidemiological profile in the countries, are required.

## Conclusions

The VL elimination strategy in Nepal provides a good example of the power of IR to facilitate disease elimination through an effective partnership of stakeholders with national disease programmes as part of a WHO regional effort. Investment by TDR and others in collaborative IR has led to a series of strategic decisions with significant impact on disease burden. This successful model of a sustained research-to-practice-to-research cycle, of country-led IR informing national decision, is rich with good practices that can potentially be replicated in other countries with diseases targeted for elimination or serve as case studies in implementation science.

**Top Five Papers**

1. Joshi AB, Das ML, Akhter S, Chowdhury R, Mondal D, Kumar V, et al. Chemical and environmental vector control as a contribution to the elimination of visceral leishmaniasis on the Indian subcontinent: cluster randomized controlled trials in Bangladesh, India and Nepal. BMC Medicine. 2009;7(1): 1–9.
2. Banjara MR, Das ML, Gurung CK, Singh VK, Joshi AB, Matlashewski G, et al. Integrating case detection of visceral leishmaniasis and other febrile illness with vector control in the post-elimination phase in Nepal. Am J Trop Med Hyg. 2019;100(1): 108.
3. Banjara MR, Hirve S, Siddiqui NA, Kumar N, Kansal S, Huda MM, et al. Visceral leishmaniasis clinical management in endemic districts of India, Nepal, and Bangladesh. J Trop Med. 2012;2012.
4. Joshi AB, Singhasivanon P, Khusmith S, Fungladda W, Nandy A. Evaluation of direct agglutination test (DAT) as an immunodiagnostic tool for diagnosis of visceral leishmaniasis in Nepal. Southeast Asian J Trop Med Public Health. 1999;30(3):583–585.
5. Younis LG, Kroeger A, Joshi AB, Das ML, Omer M, Singh VK, et al. Housing structure including the surrounding environment as a risk factor for visceral leishmaniasis transmission in Nepal. PLoS Negl Trop Dis. 2020;14(3):e0008132.
